# ACAA1 Is a Predictive Factor of Survival and Is Correlated With T Cell Infiltration in Non-Small Cell Lung Cancer

**DOI:** 10.3389/fonc.2020.564796

**Published:** 2020-10-22

**Authors:** Huiyi Feng, Weixi Shen

**Affiliations:** Department of Oncology, Shenzhen Hospital of Southern Medical University, Shenzhen, China

**Keywords:** non-small cell lung cancer, KRAS mutation, immune cell infiltration, immune checkpoint blockade, tumor metabolites

## Abstract

Non-small cell lung cancer (NSCLC) is the predominant subtype of lung cancers. KRAS mutation is the second most prevalent mutation in NSCLC. KRAS mutant cancer cells suppress the anti-tumor T cell response. However, the underlying mechanism is still unknown. Here, we analyzed the differential expression of acetyl-CoA acyltransferase 1 (ACAA1) in various types of cancers using the TIMER database and validated the results in the NSCLC cell line H1944. We silenced oncogenic KRAS by siRNA targeting KRAS^G13D^, and employed an MAPK signaling pathway inhibitor to clarify the possible regulatory pathway. Moreover, we analyzed the correlation of ACAA1 expression level with B cells, CD4^+^ T cells, CD8^+^ T cells, neutrophils, macrophages, and dendritic cells. Correlations between expression of ACAA1 and several biomarkers of mutation burden were also tested. Finally, we evaluated the prognostic value of ACAA1 in a wide range of cancers using the Kaplan-Meier Plotter Database. We found lower expression of ACAA1 in tumor tissue than in adjacent normal tissue in various cancers. This result was confirmed using a GEO dataset. Knock-down of mutant KRAS resulted in increased ACAA1 mRNA level in H1944 cells. ACAA1 mRNA level was significantly upregulated in H1944 after treatment with MAPK pathway inhibitor sorafenib, indicating that oncogenic KRAS may downregulate ACAA1 through MAPK signaling. ACAA1 was negatively correlated with biomarkers of tumor mutation burden, including BRCA1, ATM, ATR, CDK1, PMS2, MSH2, and MDH6. Conversely, ACAA1 expression was positively correlated with infiltrating CD4^+^ cells and with Th1, Th2, Treg cells in the lung tumor microenvironment. Finally, we showed that ACAA1 is a predictive factor for survival in several cancer types. In summary, decreased ACAA1 expression is correlated with poor prognosis and decreases immune infiltration of CD4^+^ T cells in LUAD and LUSC. ACAA1 also predicts T cell exhaustion in LUSC. The mechanism underlying KRAS/ACAA1 axis-mediated regulation of immune cell infiltration requires further investigation.

**Graphical Abstract:**
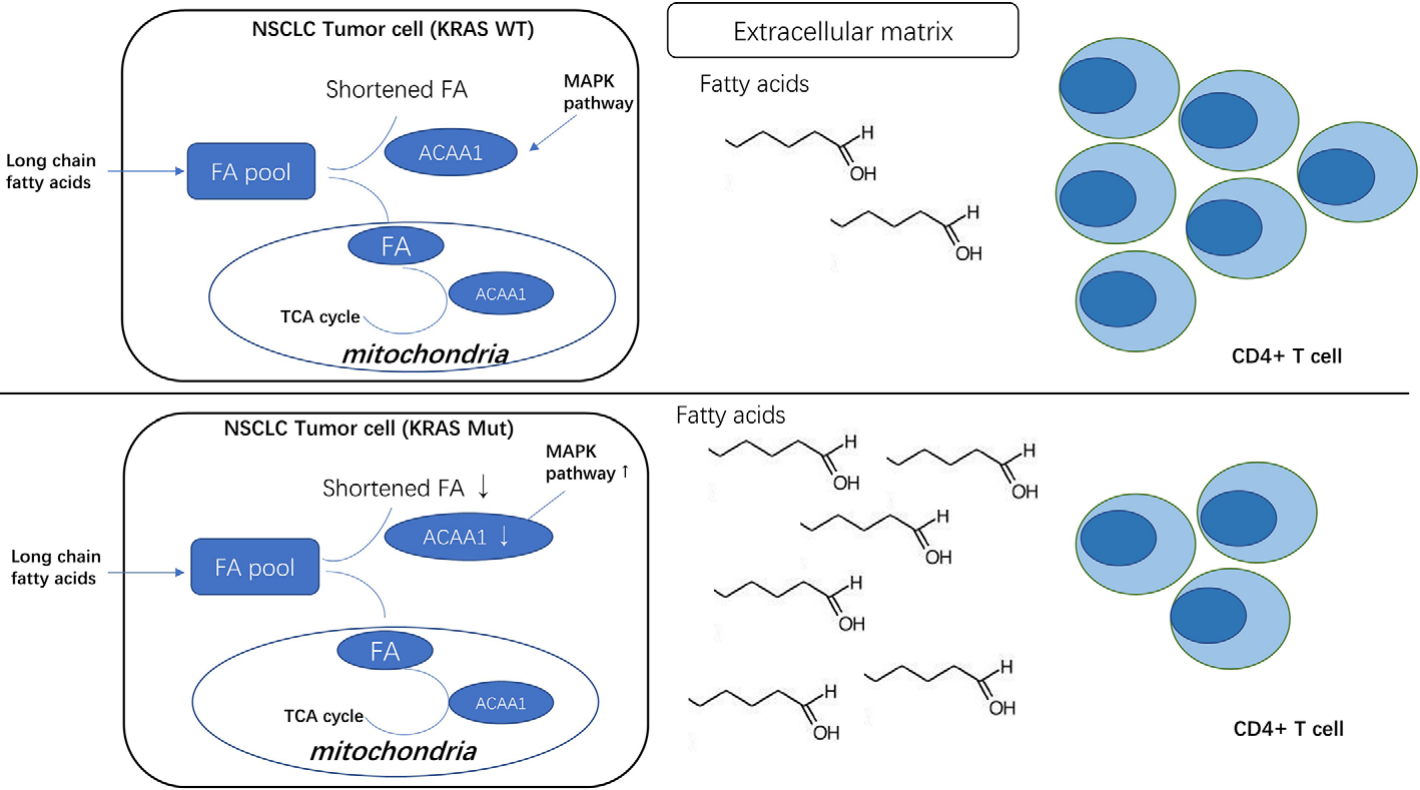


## Introduction

Lung cancer is the second leading malignancy for new cases and the first for mortality among all types of malignancies (1). Non-small cell lung cancer (NSCLC), which included lung adenocarcinoma (LUAD) and lung squamous cancer (LUSC), is the predominant subtype of lung cancer. KRAS mutation is the second prevalent mutation in NSCLC (2). Cancer cells with KRAS mutations can avoid being attacked by the immune system, facilitating immune evasion or immunosuppression phenotypes of the tumor. Immunosuppression is a basic requirement for transforming cell survival and cancer development. There are several potential mechanisms by which KRAS mutant cancer cells may suppress the anti-tumor T cell response. Among these, the ability of KRAS mutant cells to convert CD4^+^ Th cells into functional Tregs is crucial to inhibit T cell activation and promote a tolerogenic microenvironment (3). Another mechanism includes secretion of suppressive cytokines IL10 and TGFβ1 through MAPK signaling pathway (4), leading to T cell dysfunction. However, the precise mechanisms by which oncogenic KRAS induces immunosuppressive tumor microenvironment remain elusive.

By increasing the mutation burden of tumor cells, oncogenic KRAS also induces the production of a large number of neoantigens that may be recognized by CD8^+^ and CD4^+^ T cells. Specific anti-tumor T lymphocyte response can be induced after transferring KRAS mutant epitopes to adaptive T cells previously attacked (5). Tumor mutation burden is a prognostic factor for survival in a wide range of cancers (6), as well as a predictive factor for efficacy of PD-1/PD-L1 blockade immunotherapy (7).

MAPK signaling pathway is a key regulator of PD-L1 expression in lung adenocarcinoma (8). Activation of the MAPK pathway increases the expression of PD-L1 at both the mRNA and protein level, while repression of this pathway down-regulates PD-L1. Similar results were also observed in breast cancer. A preclinical study showed that combination of MAPK and PD-1 inhibitors leads to better efficacy in various types of cancers (9). Tumor cell and immune cell interaction is also regulated by MAPK pathway. In fact, MEK inhibition increases CD8^+^ T cell infiltration within the tumor, while combination of MEK and PD-1 inhibitors synergistically promotes tumor regression (10).

We noted that oncogenic KRAS suppresses the expression of acetyl-CoA acyltransferase 1 (ACAA1) *via* the MAPK signaling pathway. ACAA1 is an enzyme involved in lipid β-oxidation and provides substrates to the tricarboxylic acid (TCA) cycle, a critical step in cellular metabolism. ACAA1 is also a biomarker in type 2 diabetes (T2D), predicting the pre-diabetic metabolic signature in mouse models (11). Nwosu et al. observed that up-regulated activity of MAPK/RAS/NFκB signaling in liver cancer was associated with poor survival and identified 148 down-regulated metabolic genes regulated by the MAPK signaling pathway. These differential genes, including ACAA1, were enriched in fatty acid β-oxidation. Metabolomic studies also showed a high dependence of the tumor cells on glutamine to promote the TCA cycle (12).

Based on these scientific findings, we were motivated to analyze the potential role of ACAA1 in KRAS-mutant NSCLC and elucidate the correlation of ACAA1 with the immunosuppressive phenotype in the tumor microenvironment.

## Materials and Methods

### TIMER Database Analysis

TIMER is a comprehensive resource for systematic analysis of immune infiltrates across diverse cancer types (https://cistrome.shinyapps.io/timer/) (13). TIMER applies a deconvolution with a previously published statistical method to infer the abundance of tumor-infiltrating immune cells (TIICs) from gene expression profiles. The TIMER database includes 10,897 samples across 32 cancer types from The Cancer Genome Atlas (TCGA) to estimate the abundance of immune infiltrates. First, we analyzed differential expression of ACAA1 in pan cancer. We aimed to exclude confounding factors, such as ACAA1 expression in tumor stromal cells or immune cells. We excluded the cancer types that had no statistical significance and employed those showing statistical significance for downstream analysis. Second, we analyzed the correlation of ACAA1 expression with the abundance of immune infiltrating cells, including B cells, CD4^+^ T cells, CD8^+^ T cells, neutrophils, macrophages, and dendritic cells. Third, correlations between ACAA1 expression and markers of different T cell subsets, including Th1 cells (TBX21, STAT4, STAT1, IFN-γ, TNF-α), Th2 cells (GATA3, STAT6, STAT5A, IL13), Th17 cells (STAT3, IL17A), and Treg cells (FOXP3, CCR8, STST5B, TGFB1). T cell exhaustion markers, including PDCD1, CTLA4, LAG3, TIM-3, GZMB, were also analyzed (14). The correlation module generated the expression scatter plots between a pair of user-defined genes in given cancer types, together with the estimated statistical significance. ACAA1 was used for the x-axis with gene symbols; related marker genes are represented on the y-axis as gene symbols. The gene expression level was displayed with log_2_ RSEM. P value Spearman’s correlation coefficient was calculated automatically by the server.

### GEPIA Database Analysis

To verify the relationship between KRAS and ACAA1, we searched the expression correlation in GEPIA (15), an interactive web that includes 9,736 tumors and 8,587 normal samples from TCGA and the GTEx projects, which analyzes RNA expression. Gene expression correlation analysis was performed for given sets of TCGA expression data. We analyzed the expression pattern of ACAA1 and KRAS to identify the co-expression of several biomarkers indicating tumor mutation burden, including BRCA1, ATM, ATR, CDK1, PMS2, MLH1, MSH2, and MDH6. The Spearman method was used to determine the correlation coefficient.

### Cell Line and siRNA Transfection

Lung adenocarcinoma cell line H1944 was used to determine the regulation of ACAA1 expression. H1944 cells harbor KRAS mutation G13D. SiRNAs targeting KRAS^G13D^ (5’ GGAGGGCUUUCUUUGUGUA 3’, 5’ UCAAAGACAAAGUGUGUAA 3’), were transfected into H1944 cells using Lipofectamine 3000. One day before transfection, 25,000 cells were seeded in 24-well plates. On the day of transfection, 40 nM siRNA was diluted into 25 µl of Opti-mem, while 0.75 µl of lipofectamine was diluted in a final volume of 25 µl of Opti-mem. SiRNA and lipofectamine were mixed and allowed to react at room temperature for 10 min. After replacing complete medium with Opti-mem in each well, the total 50 µl siRNA-lipo complex was added to the wells. Twelve hours after transfection, Opti-mem medium was replaced with complete medium. Forty-eight hours after transfection, the cells were harvested, and total RNA and total proteins were extracted and used for the relevant experiments.

### MAPK Pathway Inhibition

To identify upstream regulatory pathways of ACAA1, H1944 cells were treated with the MAPK-ERK pathway inhibitor sorafenib. One day before treatment, 500,000 cells were seeded in 6-well plates. On the day of treatment, 5 µM or 10 µM of pathway inhibitor were added to the wells. The cells were collected at different timepoints (6, 12, 24, and 48 h) after treatment, and total RNA and protein were extracted using Trizol or NP40 cell lysis buffer. Subsequently, the RNA was used for q-PCR analysis.

### Kaplan-Meier Plotter Database Analysis

Kaplan-Meier plotter analyzes the correlation of RNA-seq data in over 20 cancer types with overall survival (OS) and progression-free survival (PFS) in 7,489 patients (https://kmplot.com/analysis/index.php?p=service&cancer=pancancer_rnaseq). The database is an online server indicating prognostic biomarkers in pan cancer. We searched the target gene ACAA1 in the server to identify in which types of cancer ACAA1 potentially shows prognostic value. The gene expression level cutoff was defined as 50% of RFKM, above which the expression was defined high, and conversely low. P value was calculated automatically by the server.

### Statistical Analysis

Survival curves were generated by the Kaplan-Meier plots displayed with HR and P or Cox P-values from a log-rank test. The correlation of gene expression was evaluated by Spearman’s correlation and statistical significance. P-values <0.05 were considered statistically significant.

## Results

### Expression of ACAA1 Is Lower in Tumor Tissues Than in Adjacent Normal Tissue in Various Types of Cancer

**Figure 1:**
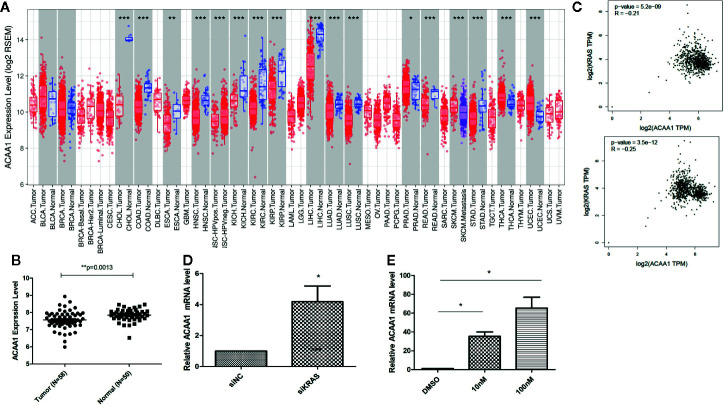
**(A)** ACAA1 expression is down-regulated in 15 types of cancer (CHOL, COAD, ESCA, HNSC, KICH, KIRC, KIRP, LIHC, LUAD, LUSC, READ, SKCM, STAD, THCA, UCEC). **(B)** The mRNA levels of ACAA1 are lower in tumor tissues than paired adjacent normal tissues (GSD 3837). **(C)** ACAA1 expression is negatively correlated with oncogenic KRAS. **(D)** Knock-down of mutant KRAS in H1944 cells results in increased ACAA1 mRNA level. **(E)** ACAA1 mRNA level is significantly upregulated in H1944 after sorafenib treatment. *p < 0.05.

First, we aimed at elucidating whether ACAA1 had different expression patterns in tumor tissue and paired adjacent normal tissue. We included all cancer types and performed the analysis in the TIMER database (**Figure 1A**). ACAA1 expression was significantly down-regulated in 15 types of cancers (CHOL, COAD, ESCA, HNSC, KICH, KIRC, KIRP, LIHC, LUAD, LUSC, READ, SKCM, STAD, THCA, UCEC), and up-regulated in only one type of cancer, PRAD. As our research interest is focused on lung cancer, we re-analyzed ACAA1 expression in tumor tissue and adjacent normal tissue using a GEO dataset (GSD 3837) (16). ACAA1 showed lower mRNA levels in tumor tissues than in paired adjacent normal tissues (**Figure 1B**). Thus, the expression pattern indicated that ACAA1 acts as a tumor suppressor in most types of cancers. (abbreviations: CHOL: Cholangiocarcinoma; COAD: colon adenocarcinoma ESCA: Esophageal Squamous Cell Carcinoma HNSC: Head-neck squamous cell carcinoma KICH: Kidney chromophobe KIRC: Kidney renal clear cell carcinoma KIRP: Kidney renal papillary cell carcinoma LIHC: Liver hepatocellular carcinoma LUAD: Lung adenocarcinoma LUSC: Lung squamous cell carcinoma READ: Rectum adenocarcinoma SKCM: skin cutaneous melanoma STAD: Stomach adenocarcinoma THCA: Thyroid carcinoma)

To analyze how oncogenic KRAS regulates ACAA1 expression and the related upstream signaling pathway, we used lung adenocarcinoma cell line H1944, which harbors KRAS^G13D^ mutation. We knocked down KRAS^G13D^ by siRNA and tested knockdown efficiency using q-PCR (**supplementary Figure 1A**). Knock-down of mutant KRAS resulted in increased ACAA1 mRNA levels (**Figure 1D**). Next, we inhibited downstream pathways of KRAS. After treatment with MAPK inhibitor, ACAA1 mRNA was significantly upregulated (**Figure 1E**). Based on these findings, we propose that ACAA1 is downregulated by oncogenic KRAS through the MAPK signaling pathway. We also confirmed the expression correlation of ACAA1 and KRAS using the GEPIA database. In LUAD and LUSC, ACAA1 expression was negatively correlated with oncogenic KRAS (**Figure 1C**).

### ACAA1 Is Negatively Correlated With Tumor Mutation Burden in Lung Cancer

**Figure 2:**
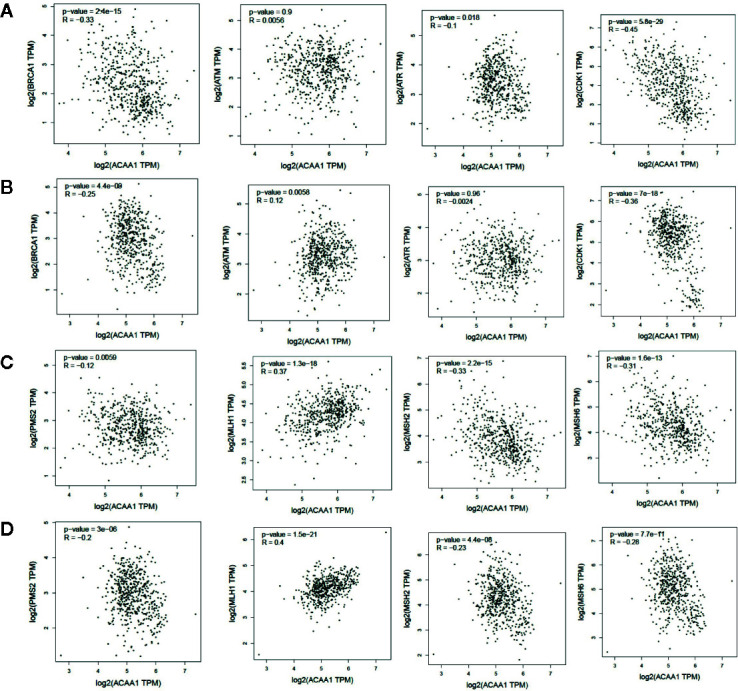
**(A, C)** In lung adenocarcinoma (LUAD), ACAA1 is correlated with BRCA1 (r= -0.33, p=2.4e-15), ATM (r= -0.0056, p=0.9), ATR (r= -0.1, p=0.015), CDK1 (r= -0.45, p=5.5e-29), PMS2 (r= -0.12, p=0.0059), MLH1 (r= 0.37, p=1.3e-18), MSH2 (r= -0.33, p=2.2e-15), and MDH6 (r= -0.31, p=1.6e-13). **(B, D)** In lung squamous carcinoma (LUSC), ACAA1 is correlated with BRCA1 (r= -0.25 p=4.4e-9), ATM(r= 0.12, p=0.0058), ATR (r= -0.0024, p=0.96), CDK1 (r= -0.36, p=7e-18), PMS2 (r= -0.2, p=3e-6), MLH1 (r= 0.4, p=1.5e-21), MSH2 (r= -0.23, p=4.4e-8), and MDH6 (r= -0.26, p=7.7e-11).

KRAS mutation is the second most prevalent mutation in lung cancer, and we observed that KRAS regulated the mRNA of ACAA1. We focused on these LUAD and LUSC in the downstream analysis. Tumor mutation burden is widely used as a biomarker for predicting efficacy of immune checkpoint blockade (17). We therefore analyzed the correlation of ACAA1 expression and biomarkers of tumor burden mutation (**Figure 2**). We found that ACAA1 was negatively correlated to BRCA1, ATM, ATR, CDK1, PMS2, MSH2, and MDH6, with statistically significant differences. BRCA1, ATM, ATR, and CDK1 are involved in DNA damage response (DDR) pathway. BRCA1 and CDK1 are the key signaling components of ATM and ATR protein kinases. Four mismatch-excision repair (MMR)-related genes, PMS2, MLH1, MSH2, and MDH6 were also tested. Except for MLH1, the other three biomarkers were negatively correlated to ACAA1. Together, these findings indicate that ACAA1 might function in maintaining DNA stability and DDR. As lung cancers harboring KRAS mutation have high response rate to PD-1/PD-L1 blockade, KRAS mutation might be a potential driver of DNA instability and DNA damage repair defects, thereby leading to the production of more neoantigens. ACAA1 might be a mediator in this process, through altering intracellular nutrients and metabolic signature.

### ACAA1 Expression Is Positively Correlated With CD4^+^ Cell Infiltration

**Figure 3:**
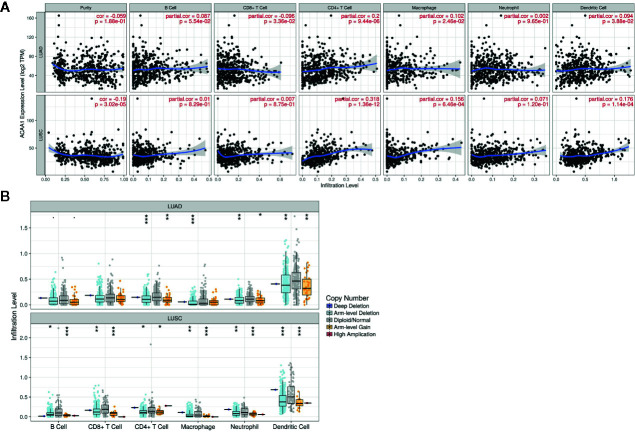
**(A)** ACAA1 is positively correlated with CD4^+^ T cell in lung adenocarcinoma (LUAD) and lung squamous cancer (LUSC) with statistical significance (r=0.2, p=9.44e-06 in LUAD; and r=0.318, p=1.36e-12 in LUSC). **(B)**. Copy number variation of ACAA1 negatively correlates with CD4^+^ cells both in LUAD and in LUSC. *p < 0.05, **p < 0.01, ***p < 0.001.

Next, we investigated whether ACAA1 expression was correlated to infiltration of immune cell in the tumor microenvironment, including B cells, CD8^+^ and CD4^+^ T cells, macrophages, neutrophils, and dendritic cells (**Figure 3A**). We found that CD4^+^ T cells were positively correlated to ACAA1 expression in LUAD and LUSC, a correlation which showed statistical significance (r=0.2, p=9.44e-06, and r=0.318, p=1.36e-12 in LUAD and LUSC, respectively). Based on this, we propose that LUAD and LUSC harboring KRAS mutation probably recruit less CD4^+^ T cells by suppressing ACAA1 expression, providing tumors with an immunosuppressive microenvironment. The insufficient number of CD4^+^ T cells in the tumor microenvironment might be due to the decrease of either T cell infiltration or polarization of CD4^+^ cells.

The TIMER database also provides a comparison of tumor infiltration levels among tumors with different somatic copy number alterations for a given gene. We confirmed that ACAA1 functionally recruited the immune cells using this feature of the database. As shown in **Figure 3B**, copy number variation of ACAA1 was negatively correlated to CD4^+^ cells both in LUAD and in LUSC. Although we do not know whether copy number alternation of ACAA1 leads to its gain or loss of function, this result partially demonstrates that ACAA1 influences immune cells infiltration in a direct or indirect manner. Taken together, the two above results show that ACAA1 was positively correlated to T cell polarization. Reduced expression of ACAA1 in the tumor cells also indicates lower level of mature T cells in tumor stroma.

### ACAA1 Is Positively Correlated With Th1, Th2, and Treg Cells in the Tumor Microenvironment of Lung Cancer

**Figure 4:**
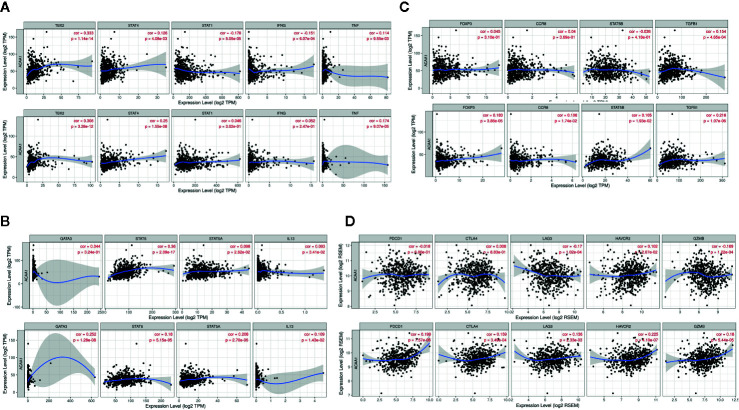
ACAA1 expression levels also positively correlate with **(A)** Th1 cell markers **(B)** Th2 cell markers **(C)** Treg cell markers **(D)**. ACAA1 is a biomarker of T cell exhaustion in lung squamous cancer (LUSC).

Further, we analyzed whether ACAA1 expression was closely related to Th1, Th2, Th17, and Treg cells by examining the expression of biomarkers from these cells. Functionally, Th1 cells facilitate differentiation of CD8^+^ cells to toxic T cells. Th2 and Th17 cells are instead negative regulators of Th1 cells. Treg cells have dual function in cancer immunology (18). These cells enable immunosuppressive cancer phenotypes by inhibiting T cell proliferation (19). FOXP3^+^ Treg is thought to promote tumor growth and metastasis by inhibiting anti-tumor immunity, and Treg accumulation in cancer is usually related to poor prognosis (20). We found that ACAA1 expression was positively correlated with TBX2, STAT4, and TNF-α, but negatively correlated with STAT1 and IFNG in LUAD, with similar expression correlation in LUSC (**Figure 4A**). As TBX2 is the main marker of Th1 cells, these results indicate a positive relationship between ACAA1 and Th1 cell infiltration in the tumor stroma. Among the Th2 cell markers (**Figure 4B**), in LUAD, ACAA1 was positively correlated to STAT6, while in LUSC, it was correlated with all Th2 cell markers, including GATA3, STAT6, STAT5A, and IL-13. STAT6 promotes naïve T cell differentiation to Th2 cells. Thus, a decrease in STAT6 in the tumor stroma results in decreased Th2 cells. ACAA1 expression levels also positively correlated with Treg cell markers (**Figure 4C**), with the best correlation in TGFB1, the major cytokine promoting T cell differentiation to Treg cells. Reduction of TGFB1 inhibits Treg cells in the tumor microenvironment. However, we found no significant correlation of ACAA1 to Th17 cells (**Supplementary Figure 1B**).

#### ACAA1 Is a Biomarker of T Cell Exhaustion in LUSC

The results presented above indicate that CD4^+^ T cell infiltration correlated with ACAA1, and most subsets of CD4^+^ cells were reduced in the tumor stroma. Subsequently, we analyzed the biomarkers indicating T cell exhaustion, to clarify the relationship between ACAA1 and this dysfunction state. We plotted the correlation of ACAA1 expression and stromal biomarkers of T cell exhaustion, including PD-1 (PDCD1), CTLA4, LAG3, HAVCR2, and GZMB. Expression of ACAA1 correlated positively with HAVCR2 and GZMB, and negatively with LAG3, showing statistical significance in LUAD (**Figure 4D**). In LUSC, ACAA1 expression was positively correlated to that of PD-1(PDCD1), CTLA4, LAG3, HAVCR2, and GZMB, with statistical significance. PDCD1 is an important biomarker of T cell exhaustion (21). Up-regulated PD-1 on T cells inhibits T cell differentiation to effector T cells and promotes T cell apoptosis. A previous study showed that oncogenic KRAS increases tumor PD-L1 expression and promotes CD8^+^ cells infiltration to the tumor stroma (22). In our study, we did not observe that KRAS mutation increased PD-L1 expression (**Supplementary Figure 1C**), nor a solid correlation of ACAA1 with PD-L1 expression in cancer cells using GEPIA (TCGA datasets) (**Supplementary Figure 1D**). More studies are needed to confirm their co-expression pattern. Nevertheless, ACAA1 seems to have different roles in LUSC and LUAD. In fact, in LUAD, ACAA1 did not show a consistent co-expression pattern with T cell exhaustion markers.

#### ACAA1 Is a Predictive Factor of Survival in a Wide Range of Cancer Types

**Figure 5:**
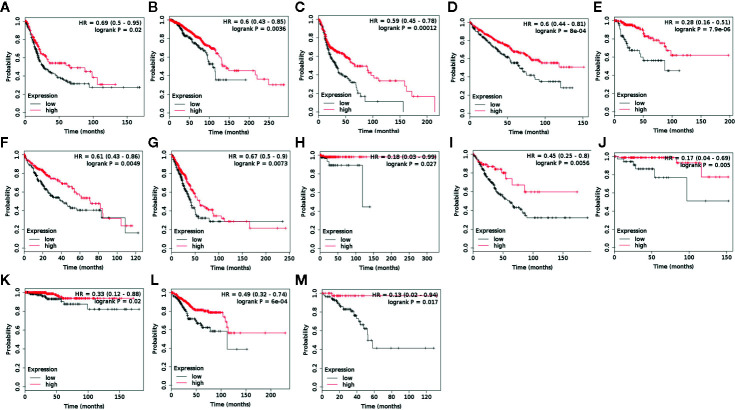
ACAA1 is a predictive factor for survival in a wide range of cancer types. **(A)** Bladder cancer. **(B)** Breast cancer. **(C)** Head-neck cancer. **(D)** Kidney renal cancer. **(E)** Kidney papillary cancer. **(F)** Liver cancer. **(G)** Lung cancer. **(H)** Pheochromocytoma and Paraganglioma. **(I)** Sarcoma **(J)** Thymoma. **(K)**Thyroid carcinoma. **(L)** Uterine corpus endometrial carcinoma. **(M)** Rectum adenocarcinoma.

ACAA1 was associated with immune cells infiltration and T cell polarization. Lastly, we investigated whether ACAA1 was a predictive factor of OS (**Figure 5A–M**) and PFS (**Supplementary Figures 2** and **3**). To this aim, we searched the survival data on Kaplan-Meier Plotter Database, which automatically generated the survival plots. ACAA1 significantly correlated with 13 out of 20 types of cancer, including bladder cancer, breast cancer, head-neck cancer, kidney renal cancer, kidney papillary cancer, liver cancer (hepatocyte carcinoma), lung cancer (lung adenocarcinoma), pheochromocytoma and paraganglioma, sarcoma, thymoma, thyroid carcinoma, uterine corpus endometrial carcinoma, and rectum adenocarcinoma. Cancers with higher ACAA1 expression level displayed higher overall survival, while those with reduced ACAA1 expression had worst outcomes. Collectively, these findings suggest that ACAA1 acts as a tumor suppressor in many types of cancers, possibly by altering the nutrient configuration and immune suppression.

## Discussion

Recent studies indicated that NSCLC harboring KRAS mutation show higher clinical response rate to and efficacy of PD-1/PD-L1 blockade (22, 23). However, the potential underlying mechanisms are still obscure. Our study suggests that oncogenic KRAS suppresses ACAA1 expression through MAPK signaling pathway. We found that ACAA1 positively correlates with CD4^+^ cell infiltration and T cell exhaustion. Moreover, reduced ACAA1 expression is associated with lower overall survival in various types of cancer, indicating that ACAA1 acts as a tumor suppressor in a wide range of malignancies. Oncogenic KRAS enhances immune checkpoint inhibitor efficacy by providing an inflammatory tumor microenvironment (24). Additionally, oncogenic KRAS also increases tumor PD-L1 expression and promotes CD8^+^ cells infiltration into the tumor stroma (22). Moreover, NSCLC harboring KRAS mutation present a higher tumor mutation burden, leading to tumor immunogenicity. Here, we found that ACAA1 was positively correlated with CD4^+^ cell infiltration. Copy number variation of ACAA1 also pointed to lower abundance of CD4^+^ in the tumor microenvironment. Although the impact of ACAA1 mutation on cancer has not been thoroughly investigated yet, these results partially demonstrate that ACAA1 functionally recruits immune cells to the tumor microenvironment.

The mean TMB and the proportion of patients with a TMB >10 or >20 mut/Mb is significantly higher for KRAS-mutated patients (10.3 mut/Mb) than for EGFR, ALK, ROS1 or MET exon 14-mutated patients (3.1 to 6.2 mut/Mb) (25). The higher mutation burden of KRAS mutant lung cancer may be due to the higher proportion of smokers among the patients presenting this type of cancer (26). However, whether the occurring KRAS mutation simply coincides with other genetic mutations or is the direct cause of the downstream DNA instability remains unknown. One potential mechanism by which oncogenic KRAS could regulate tumor mutation burden is *via* promoting fatty acid accumulation in tumor cells (27). A former study showed that oncogenic KRAS induces fatty acid synthase (FASN) to enhance lipogenesis with a specific lipid signature in lung adenocarcinoma (28). KRAS also activates ERK2 protein by upregulating ERK1. Consistently, FASN inhibition blocks cellular proliferation of KRAS-driven lung cancer cells. Importantly, saturated fatty acids play a negative role in DDR by compromising the induction of p21 and Bax expression in response to double-strand breaks and ssDNA. Moreover, saturated fatty acids appear to regulate p21 and Bax expression *via* Atr-p53-dependent and -independent pathways (28). ACAA1 transfers acetyl-CoA to fatty acids and provides substrates to the TCA cycle. Therefore, downregulated ACAA1 expression may result in accumulation of fatty acids in cancer cells, consequently leading to DNA instability.

Nonetheless, how ACAA1 mediates immune cell infiltration, as well as whether this phenomenon is causally related to ACAA1 expression or just coincidental, was still unknown. If there were a causal relationship, an appropriate explanation to it would be that metabolic shifts in tumor cells might alter the nutrient configuration in tumor stroma. Tumor cells and immune cells interact with each other through microenvironmental nutrient competition (29–31). Cancer cells utilize glycolysis as their predominant energy source, a biological phenomenon called “Warburg effect”. Nutrient competition between cancer cells and immune cells extends the function of Warburg effect to a cell-extrinsic advantage. Warburg effect promotes depletion of extracellular glucose in the tumor microenvironment, which renders tumor-infiltrating T cells dysfunctional. Glucose in the tumor stroma is reduced due to the increased glycolysis within tumor cells, which restricts glucose availability to T cells and leads to their dysfunction (32). Tumor cells produce and secrete lactic acid into the tumor microenvironment. In addition to glucose metabolism, amino acid and fatty acid metabolism also change in tumor cells. Importantly, all these metabolites affect T cell polarization. Oxidative phosphorylation (OXPHOS) and fatty acid oxidation (FAO) fuel Treg cells. Memory T cells rely on FAO, while activated Tregs primarily depend on glycolysis and fatty acid synthesis. Nutrient availability in the tumor microenvironment favors different subtypes of T cells (33). ACAA1 was recently also implicated in regulating infiltration of T cell subtypes in the tumor stroma. Yang and colleagues reported that the anti-tumor response of mouse CD8^+^ T cells can be potentiated by modulating cholesterol metabolism. They found that, in mice, inhibition of acetyl-CoA acetyltransferase 1 (Acat1) in CD8^+^ T cells restores their antitumor effect and reduces cancer progression and metastasis (34). Following CD8^+^ T cell activation, the mRNA levels of a subset of genes involved in cholesterol biosynthesis and transport pathways are upregulated, while genes implicated in the cholesterol efflux pathway are downregulated. Acat1 (ACAA1 in our study) mRNA levels are significantly upregulated at early time points, when CD8^+^ cells are activated. Moreover, inhibitors targeting Acat1 could restore CD8^+^ cell function, and a combination of PD-1/PD-L1 antibody and Acat1 inhibitor demonstrated greater efficacy than the single agents. Interestingly, Patsoukis and colleagues showed that PD-1 preserves effector T-cell function by inhibiting glycolysis and promoting fatty acid oxidation in CD4^+^ T cells through carnitine palmitoyltransferase I (CPT1A) (35). Besides predicting the efficacy of PD-1/PD-L1 inhibition, Zhang et al. reported that the ACAA1 expression level was negatively correlated to several inhibitors, including Scr inhibitor (AZD0530), MEK1/2 inhibitor (AZD6244), EGFR inhibitor (Erlotinib), HER inhibitor (Lapatinib), and VEGFR2/3 inhibitor (ZD-6474) (36). Their findings reaffirmed the important role of ACAA1in predicting therapeutic efficacy.

More studies are needed to demonstrate nutrient competition between tumor cells and immune cells. *In vitro* metabolic analysis can improve our understanding of the metabolic phenotypes specific of tumor cells and immune cells, and how they interact with each other. However, the information provided by *in vitro* studies may not be generalizable *in vivo*. The metabolic phenotype of nutrient competition relies on the supply of related fuels, such as glucose, amino acids, and fatty acids, which are certainly present at lower concentrations *in vivo* than in laboratory culture conditions. The consequence of the limited supply of these fuels in a discrete immune microenvironment in the body is likely a change in metabolic as well as nutrition-sensitive signaling pathways that affect the fate and function of immune cells. The lack of research tools for measuring nutrient distribution at the single-cell level also severely hinders our understanding of when and where nutrients are available in the body. Therefore, elucidating how nutrient supply affects immune cell metabolism and tumor-stroma interactions remains a major challenge in the field of immune metabolism.

The function of ACAA1 is to provide substrates entering the TCA cycle to fuel the cell. Suppression of ACAA1 might therefore lead to reduced activity of this cycle. The TCA cycle is a series of critical reaction used by all aerobic organisms. The main metabolic shift in solid tumors known as Warburg effect refers to the preferable use of glycolysis in cancer cells to gain fuel, instead of the more effective pathway of oxidative phosphorylation (37). By generating and secreting lactate into the tumor microenvironment, tumor cells provide acidic and hypoxic conditions to the tumor stroma and increase the nutritional pressure on effector T cells (38). Consequently, metabolites from tumor cells accumulate in the tumor microenvironment and lead to T cell metabolic switch, resulting in T cell dysfunction and exhaustion (39).

## Conclusion

Here, we showed that decreased ACAA1 expression correlates with poor prognosis and decreased immune infiltration of CD4^+^ T cells in LUAD and LUSC, and predicts T cell exhaustion in LUSC. As ACAA1 catalyzes fatty acid entry into the TCA cycle, we speculate that this phenotype could be due to nutrient competition between cancer cells and immune infiltrates. Nevertheless, clinical cohort is inevitable in validation the predictive power of this marker and the exact mechanism warrants further investigation.

## Data Availability Statement

The datasets presented in this study can be found in online repositories. The names of the repository/repositories and accession number(s) can be found in the article/**Supplementary Material**.

## Author Contributions 

WS provided the general idea. HF did the analysis and wrote the manuscript. All authors contributed to the article and approved the submitted version.

## Funding

The study was kindly granted by Sanming Project of Medicine in Shenzhen (No. SZSM201612023).

## Conflict of Interest

The authors declare that the research was conducted in the absence of any commercial or financial relationships that could be construed as a potential conflict of interest.

## Supplementary Material

Supplementary Figure 1
**(A)** KRAS^G13D^ by knockdown efficiency by siRNA using q-PCR. **(B)** No significant correlation of ACAA1 to Th17 cells in LUAD and LUSC. **(C)** KRAS mutation did not increase PD-L1 expression. **(D)** No solid correlation of ACAA1 with PD-L1 expression in cancer cells using GEPIA database analysis.

Supplementary Figure 2 ACAA1 was not a predictive factor of PFS. **(A)** Bladder Carcinoma **(B)** Breast cancer **(C)** Cervical squamous cell carcinoma **(D)** Esophageal Adenocarcinoma **(E)** Esophageal Squamous Cell Carcinoma **(F).** Head-neck squamous cell carcinoma **(G)** Kidney renal clear cell carcinoma **(H)**. Kidney renal papillary cell carcinoma **(I).** Liver hepatocellular carcinoma **(J)** Lung adenocarcinoma.

Supplementary Figure 3 ACAA1 was not a predictive factor of PFS. **(A)** Lung squamous cell carcinoma **(B)**. Ovarian cancer **(C)** Pancreatic ductal adenocarcinoma. **(D)** Pheochromocytoma and Paraganglioma. **(E)** Rectum adenocarcinoma **(F).** Sarcoma **(G)** Stomach adenocarcinoma **(H)**Testicular Germ Cell Tumor **(I).**Thyroid carcinoma **(J)** Uterine corpus endometrial carcinoma.

Supplementary Figure 4 ACAA1 was not a predictive factor of OS in the following types of cancers. **(A)** Cervical squamous cell carcinoma **(B)** Esophageal Adenocarcinoma **(C)** Esophageal Squamous Cell Carcinoma **(D)** Ovarian cancer **(E).** Pancreatic ductal adenocarcinoma **(F)** Stomach adenocarcinoma **(G)** Testicular Germ Cell Tumor.
