# Supplementary figures and images for: ACAA1 Is a Predictive Factor of Survival and Is Correlated With T Cell Infiltration in Non-Small Cell Lung Cancer
Source: Front Oncol. 2020 Oct 22;10:564796. doi: 10.3389/fonc.2020.564796 (PMC7642998; doi:10.3389/fonc.2020.564796)

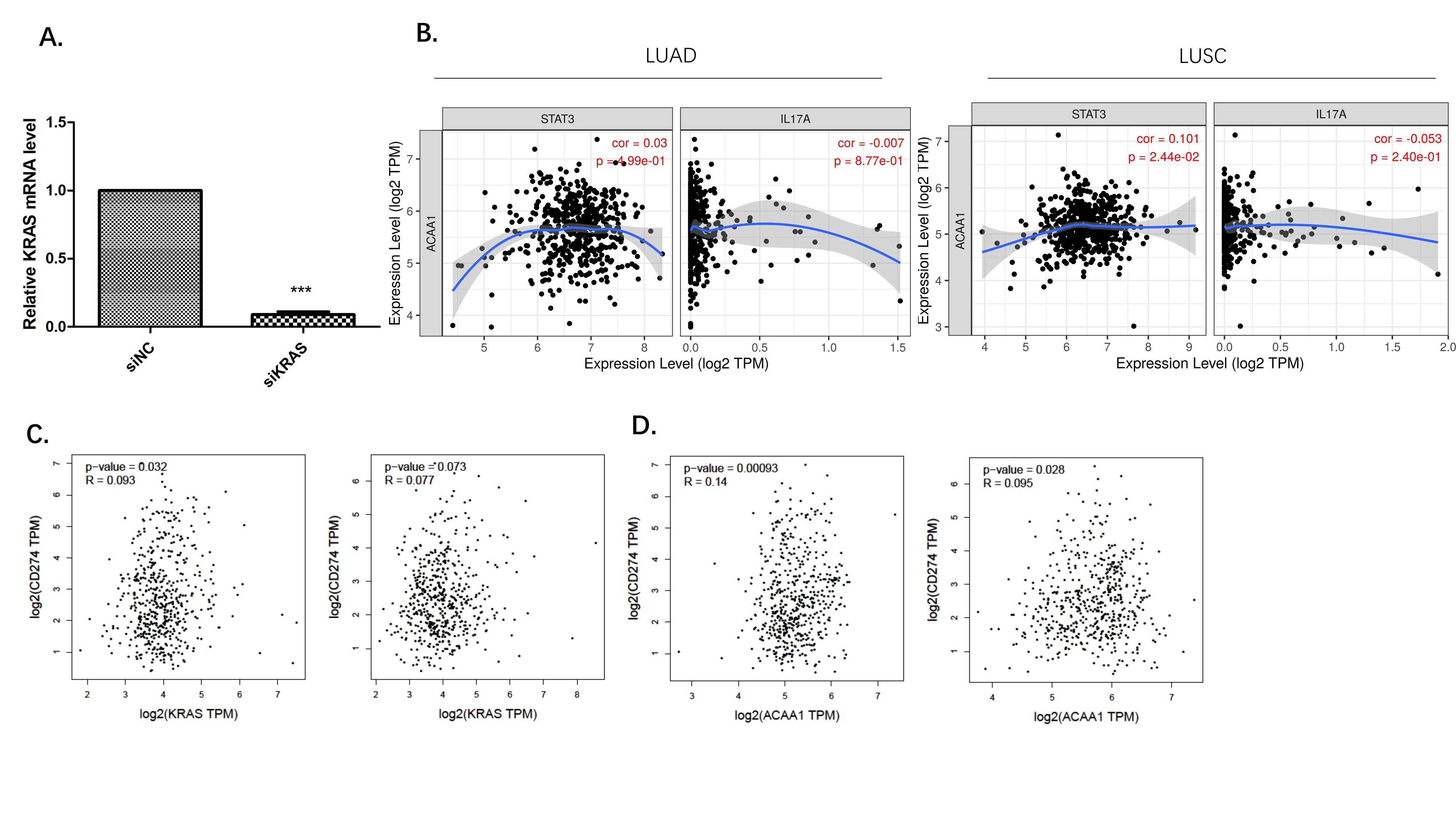

Supplement: Supplementary Figure 1 — (A) KRASG13D by knockdown efficiency by siRNA using q-PCR. (B) No significant correlation of ACAA1 to Th17 cells in LUAD and LUSC. (C) KRAS mutation did not increase PD-L1 expression. (D) No solid correlation of ACAA1 with PD-L1 expression in cancer cells using GEPIA database analysis. [file Image_1.jpeg]

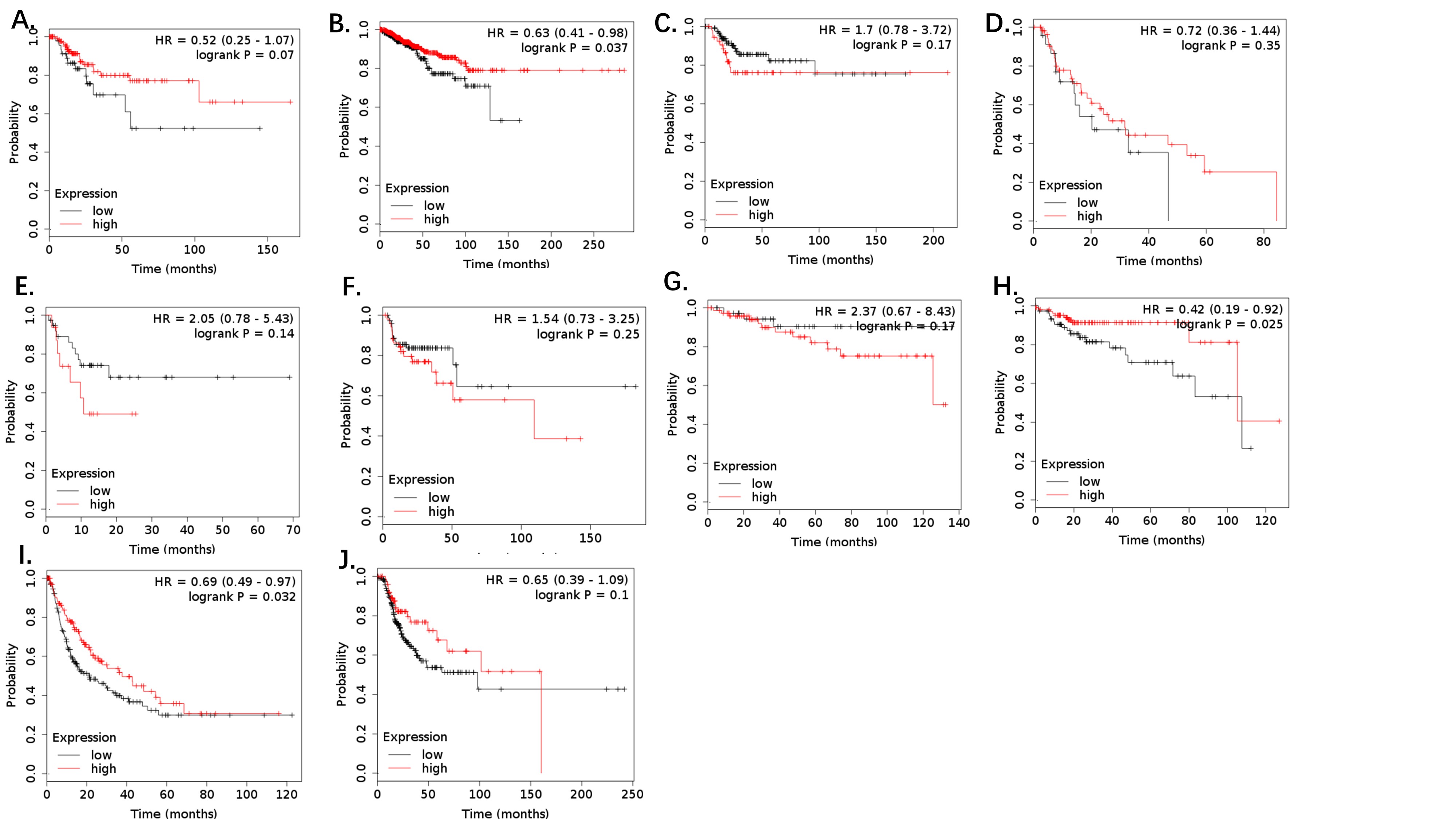

Supplement: Supplementary Figure 2 — ACAA1 was not a predictive factor of PFS. (A) Bladder Carcinoma (B) Breast cancer (C) Cervical squamous cell carcinoma (D) Esophageal Adenocarcinoma (E) Esophageal Squamous Cell Carcinoma (F). Head-neck squamous cell carcinoma (G) Kidney renal clear cell carcinoma (H). Kidney renal papillary cell carcinoma (I). Liver hepatocellular carcinoma (J) Lung adenocarcinoma. [file Image_2.jpeg]

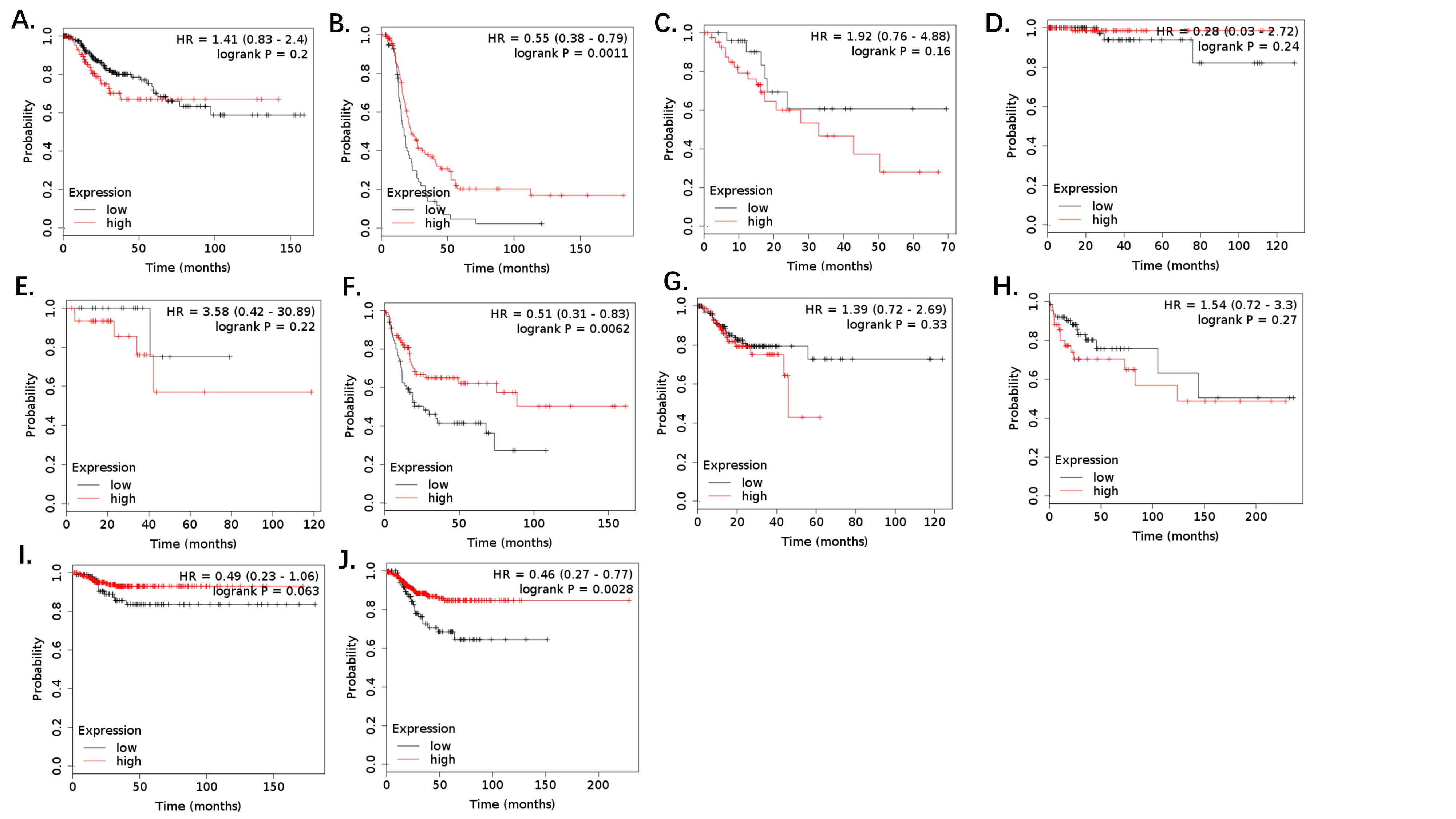

Supplement: Supplementary Figure 3 — ACAA1 was not a predictive factor of PFS. (A) Lung squamous cell carcinoma (B). Ovarian cancer (C) Pancreatic ductal adenocarcinoma. (D) Pheochromocytoma and Paraganglioma. (E) Rectum adenocarcinoma (F). Sarcoma (G) Stomach adenocarcinoma (H)Testicular Germ Cell Tumor (I).Thyroid carcinoma (J) Uterine corpus endometrial carcinoma. [file Image_3.jpeg]

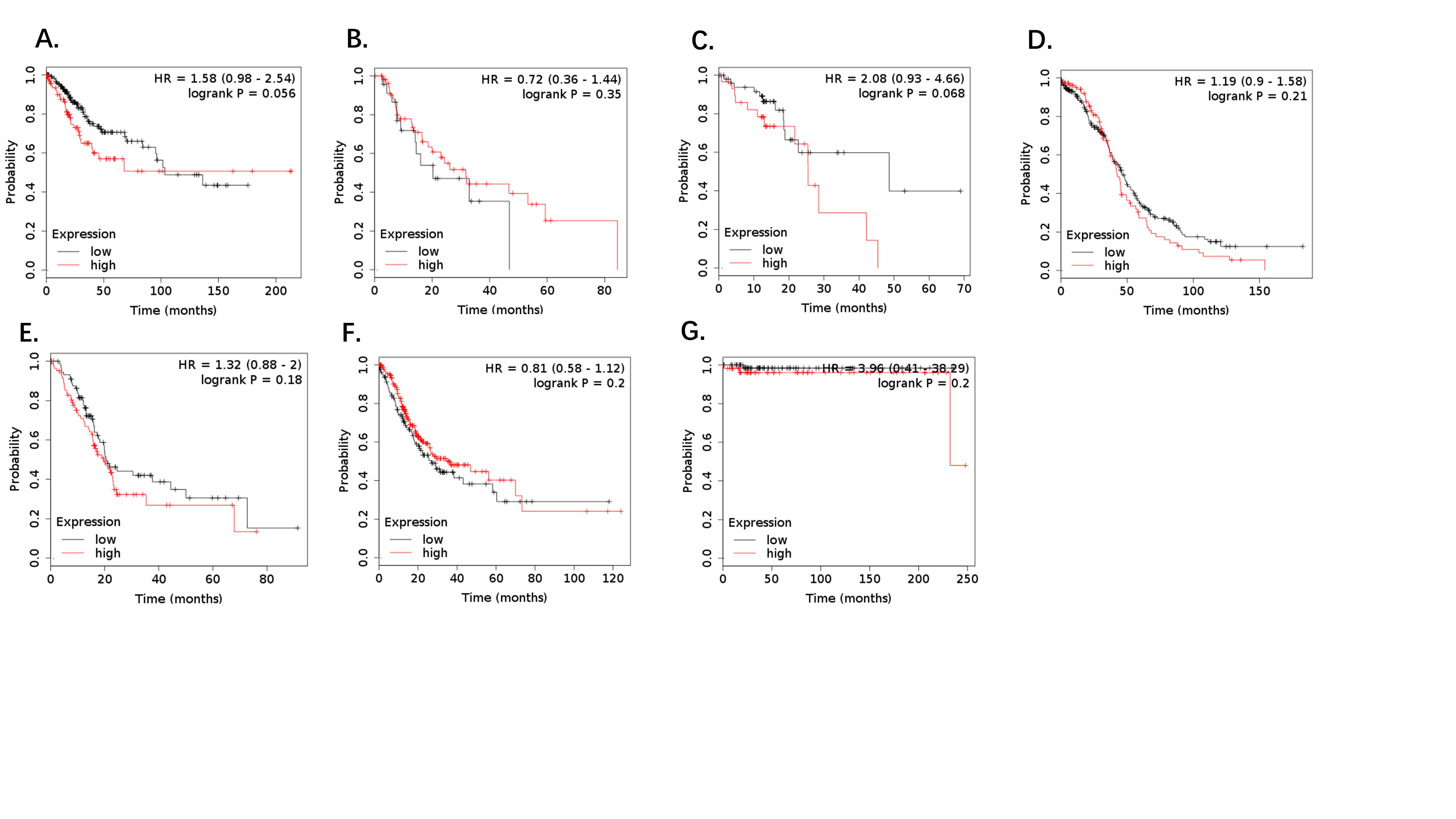

Supplement: Supplementary Figure 4 — ACAA1 was not a predictive factor of OS in the following types of cancers. (A) Cervical squamous cell carcinoma (B) Esophageal Adenocarcinoma (C) Esophageal Squamous Cell Carcinoma (D) Ovarian cancer (E). Pancreatic ductal adenocarcinoma (F) Stomach adenocarcinoma (G) Testicular Germ Cell Tumor. [file Image_4.jpeg]
